# ASSESSMENT OF DECISIONAL COMPETENCE IN DEMENTIA– A QUALITATIVE STUDY OF 60 EUTHANASIA CASES

**DOI:** 10.1093/geroni/igad104.3184

**Published:** 2023-12-21

**Authors:** Arne van den Bosch, Radboud Marijnissen, Denise Hanssen, Richard Oude Voshaar

**Affiliations:** University Medical Center Groningen, Groningen, Groningen, Netherlands; University Medical Center Groningen, Groningen, Groningen, Netherlands; University Medical Center Groningen, Groningen, Groningen, Netherlands; University Medical Center Groningen, Groningen, Groningen, Netherlands

## Abstract

The Netherlands allows euthanasia or assisted suicide (EAS) for people with dementia. The number of dementia EAS cases gradually increases every year, up to 288 cases in 2022. This practice remains controversial, as dementia is not directly lethal and may impair decisional competence. The Dutch euthanasia review committees (RTE) refer to Appelbaum and Grisso’s criteria for the assessment of decisional competence, but how these criteria are applied in clinical practice remains unknown. This study examined qualitatively which factors, and how, influence the judgment of decisional competence for EAS requests of people with dementia. Thematic analysis of 60 dementia EAS case summaries published online by the RTE between 2012 and 2021. Twenty patients had an advance directive and were decisionally compromised at time of EAS. Forty patients were decisionally competent at time of EAS, of which twenty also had an advance directive (purposive sampling). Two researchers independently coded all text related to decisional competence. A theoretical framework was developed. The four cognitive criteria of Appelbaum and Grisso were dimensional and cut-off points were influenced by six supporting factors that also directly impacted on competence assessment, i.e., level of communication, psychiatric comorbidity, personality, presence of an advance directive, consistency of the request, and the patient-physician relationship. The number of involved physicians and executed investigations were two contextual factors. The multidimensionality and subjectivity of decisional competence assessment may pose ethical and legal challenges. Continuous quality improvement processes may be needed in daily care, including possibilities for reflection.

